# The ParentingWell Practice Approach: Facilitating implementation in U.S. adult mental health services

**DOI:** 10.3389/fpsyt.2024.1377088

**Published:** 2024-07-01

**Authors:** Miriam Heyman, Joanne Nicholson, Kelly English

**Affiliations:** ^1^ Heller School for Social Policy and Management, Brandeis University, Waltham, MA, United States; ^2^ Massachusetts Society for the Prevention of Cruelty to Children, Eliot Community Human Services, Lexington, MA, United States

**Keywords:** parents with mental illness, adult mental health services, intervention adaptation, family-focused practice, recovery

## Abstract

**Background:**

To address the need for interventions for families with parents with mental illness, the evidence-based intervention Let’s Talk about Children (LTC) was adapted in the context of adult mental health services in the United States and reframed as the ParentingWell Practice Approach. This study focuses on the early implementation phase of the adapted practice in Massachusetts.

**Methods:**

As part of the adaptation and implementation process, practitioners from provider agencies serving adults with mental illness were invited to participate in the ParentingWell Learning Collaborative (PWLC), which included in-person learning collaborative sessions and follow-up virtual coaching sessions. This paper focuses on data obtained during and in response to the PWLC virtual coaching sessions, from 29 participants. Specific research questions included: (1) What themes emerged in coaching sessions related to practitioners’ experiences during the early implementation of the ParentingWell Practice Approach (2) In what ways are coaching sessions helpful to the practitioners as they implement the ParentingWell Practice Approach? Coaching sessions were recorded, and transcribed, and the data were analyzed qualitatively to identify early implementation themes. Practitioners completed feedback surveys online (which included Likert scale items and open-ended questions) following virtual coaching sessions to evaluate the usefulness of coaching sessions.

**Results:**

Coaching sessions reflected the following themes related to practitioners’ experiences during the early implementation of ParentingWell: (1) practitioners identify and share concrete approaches to supporting parents; (2) practitioners reflect on parents’ needs related to support, advocacy, problem-solving, and parenting skills; (3) practitioners reflect on their own personal experiences; and (4) practitioners’ recognize the importance of self-care strategies for themselves and for parents served. Practitioners indicated that coaching sessions were useful in supporting the implementation of a new practice.

**Conclusion:**

This study provides preliminary evidence for the benefits of coaching in the implementation of ParentingWell. Future research will explore the impact of ParentingWell on outcomes for parents and families served.

## 1 Introduction

In the United States, adults with mental illness are as likely as adults without mental illness to be parents (1). Mental illness may include anxiety disorders, mood disorders such as depression or bipolar disorder, schizophrenia, and/or other conditions. Approximately 18.2% of United States parents of children under the age of 18, equivalent to roughly 12.8 million individuals, experience mental illness (2).

Parenthood is a salient component of one’s identity, and yet many parents with mental illness contend with societal assumptions that mental illness and parenthood are incompatible, and that they are dangerous to themselves and to their children (3). Societal stigma and the notion that parents with mental illness are inherently incompetent contribute to disproportionately high rates of child welfare system involvement and related outcomes including loss of child custody for parents with mental illness (4–9). These outcomes can lead parents to perceive that they have failed in their parenting roles, and thus be deleterious for their mental health and wellbeing, exacerbating existing mental illness (3, 5).

Despite these challenges that confront parents with mental illness, behavioral health practice remains largely focused on the individual, without explicit attention paid to that individual’s family circumstances (including whether they currently live with their children, are estranged from their adult children, are currently engaged with child welfare agencies, etc.), and how these circumstances intersect with their wellbeing (10–17). This is a critical gap, since a focus on parenting can provide motivation for recovery, while the stresses of parenting, if not explicitly acknowledged and addressed, can be detrimental to wellbeing (18). The potentially negative impact of parental mental illness on children, especially in the context of inadequate support for parents, and the general interconnectedness between parent and child wellbeing, further demonstrates the need to address extant gaps in behavioral health practice (19).

Mental health practitioners (e.g., psychologists, social workers, psychiatric nurses, counselors, case workers, peer specialists) may lack the relevant skills, knowledge, and confidence to adequately address parenting and family considerations with their adult clients who are parents (10, 20–26). Gaps in practitioners’ skills, knowledge, and confidence may impact their ability to effectively serve parents, who may present unique challenges in accessing care (i.e., child care, concerns around mandated reporting, etc.) (10, 20–26). Additionally, there are systems-level or contextual barriers to implementing parent and/or family-focused behavioral health practices. These contextual barriers include practitioners’ perceptions of workplace support or lack thereof, the need to invest time and other resources to implement new practices, and the need for ongoing supervision related to new practices (22, 24, 27). As such, the larger ParentingWell research program aims to address the gap in parent and family-informed practice in mental health services in the United States, through the process described in the following paragraphs.

The ParentingWell Practice Profile (28) was developed in the context of a state-wide initiative to adapt the evidence-based intervention Let’s Talk about Children (LTC). LTC was developed in Finland (29–33) and replicated and tested in Australia (26, 34–36) Greece (37) China (38) and Japan (39). It aims to promote optimal parenting and child development and prevent children’s mental health problems by providing their parents with information and opportunity to discuss their children. To implement LTC, providers are trained to use a semi-structured interview tool during three or four prescribed sessions, during which parents are encouraged to discuss the child, the parents’ mental illness and its intersection with family life, plans to promote child and family wellbeing, and available services and supports (31).

The process of adapting LTC in Massachusetts occurred between 2015 and 2019 and included: (1) consulting with the LTC purveyor to specify the core elements and theories underlying the initial intervention; (2) consulting with key stakeholders regarding the Massachusetts target population and service context; (3) pretesting initial adapted materials; and (4) making iterative refinements to compile the final product, the ParentingWell Practice Profile (40). The resultant product, The ParentingWell Practice Profile, differs from the original intervention in that it is an approach designed to be integrated into existing routines, as opposed to being a prescribed stand-alone intervention. It includes tools and conversation topics intended to support practitioners to conduct family-focused conversations, generate a family-informed service plan, or provide services to adults living with a mental health condition or addiction, while maintaining engagement around parenting and family circumstances. It is applicable for diverse practitioners and settings, addresses parenting across the life span, and builds on practitioners’ existing skills (e.g., motivational interviewing). Consistent with Self-Determination Theory, it consists of four core elements (engage, explore, plan, access and advocate) and four underlying principles, drawn from LTC (trauma-informed, strengths-based, family-focused, culturally sensitive).

The ParentingWell Learning Collaborative (PWLC) aims to prepare and support mental health practitioners in implementing ParentingWell (41). It consists of training and debriefing sessions, virtual coaching sessions, and access to an interactive online hub. Existing research demonstrates support for the feasibility of the PWLC. Participants in the pilot were highly engaged in and satisfied with the PWLC, and they utilized PWLC skills, tools, and resources (40). Thus far, this body of work pertaining to the adaptation of LTC, the resulting ParentingWell Practice Profile (28), and PWLC represents the exploration and preparation phases of the dynamic adaptation process to implement an evidence-based intervention (42). The dynamic adaptation process is specified to facilitate the strategic and planful adaptations of interventions in the community, organizational, and service system contexts in which they will be implemented (42). Within the preparation and exploration phases, Adaptation Team members gather information pertaining to system and organizational contexts (i.e., payment procedures, paperwork requirements, etc.) as well as provider and client characteristics (42). The adaptation activities that have been described elsewhere, including, and resulting in the specification of the ParentingWell approach in collaboration with key stakeholders, align with these phases (40, 41).

The training of practitioners and the provision of support “for intervention, system, and organizational adaptation and intervention to meet local needs” falls within the implementation phase (42). At the same time, the implementation phase can address issues including permissible adaptations to the model and questions related to model fidelity (42). Practitioner retention and satisfaction are critical components of the sustainment phase (42). In order for practitioners to sustain the intervention, they must be satisfied with its usage and retain it accordingly. Similarly, Movsisyan et al. (2019) delineate the same four steps for intervention adaptation: exploration, preparation, implementation and sustainment (43). They identify routine, ongoing supervision for trained staff and evaluation of the model as key components of the latter two phases. Coaching sessions are thus a vehicle for successful implementation – a prerequisite for evaluating impact.

As such, this study focuses on the implementation of the adapted ParentingWell Practice Approach. Our specific research questions are as follows: (1) What themes emerged in coaching sessions related to practitioners’ experiences during the early implementation of the ParentingWell Practice Approach? (2) In what ways are coaching sessions useful to the practitioners as they implement the ParentingWell Practice Approach? Themes related to the implementation of ParentingWell will illuminate how practitioners are utilizing ParentingWell; this implementation information is a critical prerequisite for subsequent evaluations of efficacy and effectiveness. We also aim to explore the coaching sessions as a mode of providing ongoing support, which is integral to the dynamic adaptation process, facilitating implementation and sustainment (42).

## 2 Materials and methods

The process of adapting LTC into ParentingWell took place between 2015 and 2019, and included the following steps: 1) consulting with the creator of LTC to identify the underlying core elements and theories (2); consulting with key stakeholders in the Massachusetts behavioral health service context regarding characteristics of this context and its’ target population (3); pretesting initial adapted materials and (4) making iterative revisions to yield the final product, the ParentingWell Practice Profile (40).

In March of 2019, participants from all provider agencies in Massachusetts serving adults with mental illness were invited by the Massachusetts Department of Mental Health to participate in the ParentingWell Learning Collaborative (PWLC) (41). Agencies identified as mental health service vendors by the Massachusetts Department of Mental Health received an invitation via email to send three to five staff members to the PWLC. At least one staff member had to be a supervisor or program manager, and this person was responsible for agency-wide implementation of ParentingWell. Participating staff members had various roles (i.e., clinicians, peer specialists, case managers), and were required to be located at a single site and work within a single program area or care level. Practitioner participants were required to identify current parents served (or adults served who were planning to become parents), and each agency identified a senior leader to sponsor the initiative (e.g., a CEO or Executive Director). Following project team review of agency applications, 30 participants representing five agencies were selected to participate in the PWLC. Additional information about the recruitment and selection process is available elsewhere (41).

The PWLC included the following components: an orientation session for each agency (held onsite at each participating agency); three in-person, full-day learning collaborative sessions held in May and June of 2019; a virtual project hub which served as a space for participants to access logistical and supplemental information and resources; coaching sessions for four months following the conclusion of the three learning collaborative sessions, and a debriefing session in November of 2019. Each component is described in more detail elsewhere (41). The coaching sessions occurred in July through October of 2019. They were offered at four different times each month; practitioners were encouraged to attend one session per month (a total of four sessions per participant). Each coaching session was one hour and was held via video conferencing. During each coaching session, facilitators focused on one core element from the ParentingWell Practice Profile (i.e., Engage, Explore, Plan, Access & Advocate). Participants were encouraged to share related experiences, insights, successes, challenges, and tips and resources.

Participants completed an initial background and demographic survey prior to the start of the in-person learning collaborative sessions, and attendance was tracked at each learning collaborative, coaching, and debriefing session. Coaching session participants completed semi-structured feedback online surveys (satisfaction surveys) following each session. To address the research questions, we used an exploratory, qualitative design. These methods are well-suited given the exploratory nature of our study, which aimed to develop a preliminary base of knowledge in an unexplored area (44, 45). Coaching sessions were recorded, transcribed, and analyzed with Dedoose software (46), using a framework approach (47, 48). ParentingWell Practice Profile core elements and Theory of Planned Behavior constructs provided the initial codes. Analyses were conducted by trained, experienced, doctoral level research team members. These team members met regularly to review, discuss, and agree upon code assignments and findings. For the satisfaction surveys, responses to Likert items were analyzed quantitatively; text responses regarding sessions and information shared were analyzed qualitatively.

## 3 Results

### 3.1 Participants characteristics and attendance

Twenty-nine participants completed the initial demographic and background survey, and participated in subsequent learning collaborative activities. The majority of these participants were female (75.86%) and White (93.10%). Many of the participants were parents or expected to become parents in the future (72.41%), the majority (58.62%) held a Master’s degree or higher, while 82.75% held at least an Associate’s Degree. Two thirds of participants held positions at community-based human service agencies, and they represented a wide range of job tenure and diverse roles. Peer specialists represented 31% of participants.

Attendance was consistently high across each learning collaborative in-person training session. Excluding agency executives who were not required to attend each session, total attendance at all 3 in-person learning collaborative sessions was 93% (41). Coaching sessions, conducted virtually and offered at 4 different times in each of 4 months, were less well attended. Twelve practitioners attended during the first month, eleven during the second month, 1 during the third month, and 5 during the fourth and final month (see **Table 1**).

**Table 1:** PW coaching session satisfaction survey results.

Survey Item*	Engage mean (range) *n* = 12 in 4 sessions	Explore mean (range) *n* = 11 in 3 sessions	Plan mean (range) *n* = 1 in 1 session	Access and Advocate mean (range) *n* = 5 in 3 sessions
1. I am satisfied with the format of the coaching session.	5.75 (4 – 7)	5.91 (5 – 6)	6.00	6.00 (6 – 6)
2. I found the coaching session to be applicable to my role.	5.67 (4 – 7)	5.91 (5 – 7)	6.00	5.80 (5 – 6)
3. I am satisfied with the trainer(s) who led the coaching session.	6.33 (5 – 7)	6.55 (5 – 7)	7.00	6.60 (6 – 7)
4. I am satisfied with my overall experience at the coaching session.	5.75 (3 – 7)	6.09 (5 – 7)	6.00	6.20 (6 – 7)
5. The balance between presentations, discussion and activities fits my style of learning.	5.83 (4 – 7)	6.00 (5 – 7)	6.00	5.80 (5 – 6)
6. I would recommend the coaching session to other behavioral health practitioners.	5.67 (4 – 7)	5.82 (4 – 7)	6.00	6.40 (6 – 7)

*Responses to Likert-type items ranged from “1 = strongly disagree” to “7 = strongly agree” with “4 = neither agree nor disagree”.

### 3.2 What themes emerged during coaching sessions related to practitioners’ experiences during the early implementation of the ParentingWell Practice Approach?

With regard to the first research question pertaining to themes related to early implementation experiences, the following themes emerged during coaching sessions: (1) practitioners identify and share concrete approaches to supporting parents; (2) practitioners reflect on parents’ needs related to support, advocacy, problem-solving, and parenting skills; (3) practitioners reflect on their own personal experiences; and (4) practitioners’ recognize the importance of self-care strategies for themselves and for parents served. Each theme will be explored in the paragraphs that follow.

During coaching sessions, practitioners *shared concrete approaches* to their work with parents, and concrete ideas for implementing ParentingWell in their respective settings. Practitioners mentioned specific and applicable conversation topics they utilized and found helpful in their work with parents. One practitioner shared,

“*So you can have a conversation, not about self-care, but about sort of what recharges you or what makes you feel like you have more energy. Imagine having that conversation with the person who’s depressed*.”

Another stated that she and a client who was a parent “*started tracking joy*.” A third practitioner reported developing a concrete plan with a mother for her children in the event of her hospitalization:

“*And if I go to the hospital, what happens, you know. So we talked about Plan A and Plan B and Plan C if we need that*.”

Furthermore, in addition to concrete strategies for use with parents, participants shared ways in which they will encourage and implement family-focused approaches at their agencies. For example, participants described the development of staff training and speakers’ bureaus (i.e., speakers with lived experience who would be willing to share their stories).

Importantly, coaching sessions also demonstrated ways in which practitioners *reflect on parents’ needs* related to support, advocacy, problem-solving, and parenting skills. Notably, practitioners recognized that building rapport with clients is a prerequisite to adequately addressing their needs. This rapport includes a comfort to talk about parenting, and a comfortable space in which parents can reflect on their circumstances. One practitioner described,

“*That’s why I didn’t start right out of the gate with the paperwork, because I like to get them to feel comfortable before they’ll engage in this because if you don’t have some sort of comfort level with them, they’re not going to do this*.”

This same practitioner then agreed with the following statement from a different participant:

“*Well, that’s all the work around engagement*”.

In the context of rapport, practitioners described how they perceive parents’ needs and how they work to address these needs. One participant shared that she was working on building a support group for parents, and another shared that she arranged an opportunity for two mothers to take their children to a fast-food restaurant (McDonald’s). She shared,

“*At one point, we took them like to this little play area and they were holding hands, and it was super cute and they got along pretty well. And both of the moms are open to trying it again because sometimes even as a parent, having friends can be challenging*.”

A second participant described working with a mother to become assertive without being aggressive, in order to advocate for herself and her children. Related to needs around problem-solving and parenting skills, practitioners worked with parents to develop boundaries, with adult children, for example. One practitioner shared,

“*I know she [the client/parent] can do it, but she’s not practicing the follow-through and now to distance herself and give him the independent sort of approach, saying like when you keep enabling him this way you’re denying that gift we can give him that’s free [his independence]. And she is able to do it in the moment and talk about in a moment and reflect on it, but she hasn’t been able to follow through on anything, but I, I understand that*”.

As practitioners considered ways to address parents’ needs, they emphasized the importance of leverage parents’ existing strengths and developing specific, actionable, and measurable goals to address needs. Pertaining to parents’ strengths, one participant shared,

“*So yeah, I don’t know, just sort of a cool experience to go through that checklist with her because I think she, like I said, realize that she’s closer, you know, she’s able to do some of these things, more so than she thought she could do*.”

This person was referring to a checklist that is designed to help parent clients review their situations and circumstances, identify their strengths, and set goals and priorities. From the perspective of this coaching participant, it was especially consequential for a parent as it helped her to recognize her abilities. Another participant described the utility of this exercise as she said,

“*As a way for people to write themselves, not in terms of, like, oh, I’m very good or not very good, but rather able to identify strengths that they have, but also maybe some things they want to work on.*”

Importantly, as practitioners develop strengths-based goals with parents, their work reflects the premise that goals should be specific and measurable. For example, one practitioner reported using the daily log with a parent client to develop routine and structure following separation from her partner:

“*And also not having her partner available anymore is going to mean that the weekends might be about how to make sure that there is as much structure as she can. You know, especially for kids that are going from having different routines and how her and her partner going to try and align in terms of, you know, bedtime is going to be the same at mom’s house…*”

This log allows the identification and tracking of specific and measurable goals (i.e., a consistent bedtime).

Thus, as coaching sessions provided insight into practitioners’ experiences with parents during the early implementation of ParentingWell, coaching sessions also demonstrated ways in which practitioners *reflect on their own personal experiences* as they relate to family life and recovery. One participant shared,

“*This is something that I struggle with on my own too. My son he’s going to be 19 so he’s still really young, but he doesn’t live with me because it never worked out, it just didn’t work out so he lives with a friend. But I constantly do everything for him because I don’t want to see him fail*.”

Another participant disclosed,

“*I mean, for me, unfortunately, I had to kick my son out of the house. But when it came down to it, it took him affecting my own mental wellbeing and security in my home. And then I was able to do it but I honestly believe all of those things the therapist said along the way. Just reassuring me and saying things like, When is it going to be time for you?*”

Another practitioner reflected,

“*I would love to be part of anything like that I as part of my training I shared my own experiences of raising a daughter who had some significant mental health challenges. And all of the ways that I had to support her and navigate the service system and find her supports and she’s 30 something successful young woman*”.

Furthermore, practitioners utilized coaching sessions to brainstorm and *share self-care strategies for themselves and for clients*. One practitioner shared,

“*I do meditation and I walk every day*.” A second practitioner described, “*Know somebody told me once that when you are having a bad day, get a roll of quarters and put them in a bunch of parking meters and keep watch. Wow. I love that. What a small attainable concrete way to feel like you just made a difference*.”

These quotes convey that coaching sessions were spaces for practitioners to identify and share strategies for self-care. Regarding self-care for parents served, one practitioner/participant explained,

“*We had a discussion about this notion of self-care - that self-care doesn’t mean selfish, and that lots of times when you talk with people, particularly parents about self-care, it does conjure up the notion of being selfish or that by talking time for yourself you’re taking time away from your children. Someone suggested that notion of using that daily log form as a way for people to make note of pleasurable moments during the day.*”

The daily log form is a tool within the portfolio of ParentingWell materials that helps parents recognize their multiple contributions to their children over the course of a day, as well as identify opportunities to care for themselves. Another participant described her approach with a parent client as she said,

“*Look over the course of the day and see just how much time and effort is being spent on taking care of others versus oneself. Not that you could ever achieve 50/50 or whatever but even if you could look and say, okay, an average day you spend 12 hours taking care of other people and 10 minutes taking care of yourself. Is there any way we could increase that to 15 minutes?*”

In this instance, the daily log enabled the practitioner to suggest a concrete goal for a parent client with regard to self-care.

### 3.3 In what ways are coaching sessions useful to the practitioners as they implement the ParentingWell Practice Approach?

Practitioners indicated high levels of satisfaction with the coaching sessions. Following each coaching session, the majority of participants agreed or strongly agreed with each of the following statements: I am satisfied with the format of the coaching session; I found the coaching session to be applicable to my role; I am satisfied with the trainer(s) who led the coaching session; I am satisfied with my overall experience at the coaching session; The balance between presentations, discussion, and activities fits my style of learning; I would recommend the coaching session to other behavioral health practitioners. **Table 1** displays mean responses and ranges for each statement, for each session.

In addition to the Likert scale satisfaction questions, following each coaching session, participants responded to the following open-ended prompts: What did you like about today’s session? What did you learn during today’s session? What would you change about today’s session?

In response to the first two questions, participants frequently reported that they appreciated hearing different perspectives and sharing ideas related to the real-life scenarios pertaining to their work with parents, including ideas related to ParentingWell resources. One person shared,

“*Listening to others share their experience gives me different perspectives as I work with persons served*.”

Another person said,

“*I liked the diverse perspectives being utilized*.”

Notably, as a result of these conversations, practitioners gained concrete suggestions for their own practice, and for their own agencies. One practitioner explained,

“*I learned that even though it might be easier sometimes to just do things for our families, it is better for them if you also teach them how to do it on their own. That doesn’t mean you can’t lend a helping hand when someone is overwhelmed*.”

A second practitioner shared,

“*I received helpful feedback about my goal writing. It was useful to continue to talk about the process.*”

A third practitioner reported that it was helpful to learn

“*how agencies are incorporating parenting issues into their everyday practices and following up with staff in supervision*.”

Practitioners also shared that coaching sessions provided an opportunity to enhance their ability to effectively use ParentingWell tools and resources. One practitioner said,

“*By listening to the other clinicians, I was able to learn different ways to present the materials with the people I work with*”.

Alongside this positive feedback, practitioners identified ways in which coaching sessions might be improved in the future. The coaching sessions were held virtually (to eliminate commuting time) and occurred prior to the COVID-19 pandemic. Many practitioners expressed their preference to meet in person (“*I find it easier to meet face-to-face*”; “*I still prefer to meet in-person*”; “*I understand the logistics of using Zoom, but it limited the feedback and I did not find it as effective as meeting face-to-face*”). Taken together, while practitioners responded favorably to coaching sessions, the pre-pandemic context seems to include a consistent preference for in-person interaction.

## 4 Discussion

This study explores the implementation of the adapted ParentingWell Practice Approach. We analyzed data from practitioner coaching sessions, including transcripts of coaching sessions and practitioners’ evaluations of the coaching sessions. These sessions are contexts in which trained practitioners received ongoing supervision pertaining to ParentingWell’s implementation. This post-training supervision has been identified as a key component of the final phases of intervention adaptation and implementation (44).

Data from coaching sessions provide insight into practitioners’ experiences during the early implementation of ParentingWell. This insight yields important information about actual practice, which is a prerequisite to rigorous testing efficacy and effectiveness. Our findings demonstrate that practitioners identify and utilize concrete approaches to supporting parents, they reflect on parents’ needs related to support, advocacy, problem-solving, and parenting skills, they reflect on their own personal experiences relevant to family life and recovery, and they recognize the importance of self-care strategies for themselves and for parents served. The opportunities to share personal experiences during coaching sessions are likely integral to the wellbeing and professional effectiveness of practitioners. Practitioners each have unique and salient experiences related to their parents and/or their children; these experiences inform their outlooks and thus their interactions with parent clients. Dialogue around these issues can facilitate exploration and insight related to these intersections between personal and professional experiences. This might be particularly relevant for peer specialists; future research can explore whether coaching sessions have a unique usefulness for clinicians with relatively less formal training.

Notably, many of the themes that emerged overlap with the core elements of the approach (26). These core elements include engage (building rapport and asking non-judgmentally about family life), explore (asking how things are going in the family and how they relate to recovery), planning (identifying goals related to parenting), and access and advocating (identifying supports to facilitate goal achievement). For example, in conversations about problem solving, practitioners addressed the development of measurable goals (i.e., maintaining a consistent bedtime) – a key component of the plan element. Practitioners also described building rapport with parents, and how this is an essential prerequisite to addressing parents’ needs – and this aligns with the engage element.

Practitioners also reported a high level of satisfaction with the coaching sessions, and they were able to articulate many benefits that they accrued from participation, including sharing ideas, hearing diverse perspectives, and learning how to use ParentingWell tools and resources. Taken together, the findings suggested that the coaching sessions were sources of support for practitioners, and thus an essential component of the adaptation process (43).

Critically, while this research illuminates key aspects of the implementation phase of the adaptation of LTC into ParentingWell, additional research is needed to investigate the extent to which other aspects are addressed. Specifically, Movsisyan et al. (43) highlight evaluation as critical to sustainment, while Aarons et al. (42) note that client satisfaction and retention are prerequisites for the successful completion of an adaptation process. As such, future research should explore the impact of ParentingWell on outcomes for parents and families served. This will be key to the evaluation of the approach. Findings from the current study support the notion that practitioners incorporate elements of the approach into their routine work. Additional research should seek the perspectives of parents and family members. Ultimately, given the interdependence between parental and child wellbeing, future research can explore whether and how ParentingWell facilitates wellbeing of multiple family members, including parents served and their children.

It is also important to acknowledge that the learning collaborative approach required a significant investment of time; both trainers and staff from participating agencies contributed large quantities of time to the learning collaborative and the subsequent coaching sessions. To offset some of the costs associated with lost productivity, participating agencies received a stipend as compensation for their staff’s involvement (with the exception of one agency with policies that precluded a stipend). While the coaching sessions have already been conducted virtually, practitioner feedback indicated a strong preference for face-to-face interaction. It is possible that future learning collaborative sessions can also be held virtually, to mitigate costs and travel time; it will be critical to consider costs and benefits associated with a shift towards more online content. Future research should also consider how to adapt content so that it is more suitable for an online space; this may ultimately facilitate participation of a greater number of agencies and practitioners across the United States.

This study has limitations that are important to note. The participating practitioners were part of a pilot study of the ParentingWell Learning Collaborative (41); as such the sample was small and does not permit the systematic investigation of differences according to type of practitioner or other characteristics (e.g., of setting or clients/parents). The data did not provide information about how challenges or opportunities might vary across these characteristics, and the lack of nuanced analysis is an important limitation. Also, while the sample was diverse with respect to professional characteristics such as job title and education history, the sample was predominantly White and female (93% and 76% respectively). This is likely reflective of the population of Massachusetts behavioral health providers in community-based settings. However, it is problematic given the great extent to which gender and culture individually and interactively influence parenting. To competently address family life in behavioral health settings, the workforce must be diverse and culturally competent. Future research should explore each component of the adaptation process as it pertains to ParentingWell, with practitioners and clients/parents who reflect this diversity. This aligns with the general need for implementation researchers to iteratively check acceptability, fidelity, and feasibility of interventions across multiple contexts (49).

Despite these limitations, this study provides insight about practitioners’ experiences during the early implementation of ParentingWell, and evidence for the usefulness of coaching sessions in supporting their practice. Currently, while the disparities that confront parents with mental illness are well-documented, their needs are infrequently addressed within behavioral health service systems. The development of empirically and theoretically sound practice approaches, that are accessible and useful to diverse practitioners in a range of settings, is a critical step towards addressing this gap. An emerging body of evidence pertaining to the adaptation of LTC into ParentingWell suggests that ParentingWell can comprise one of these sound practice approaches. As such, ParentingWell is a step in the right direction for parents with mental illness and their families, and the practitioners who support them.

## Data availability statement

The datasets presented in this article are not readily available. Given the qualitative nature of the data and the concern for the privacy of participants, many of whom are agency leaders, data are held by authors. Requests to access the datasets should be directed to MH, miriamheyman@brandeis.edu.

## Ethics statement

The studies involving humans were approved by Brandeis University Human Research Protection Program. The studies were conducted in accordance with the local legislation and institutional requirements. The participants provided their written informed consent to participate in this study.

## Author contributions

MH: Conceptualization, Data curation, Methodology, Writing – original draft, Writing – review & editing. JN: Funding acquisition, Methodology, Supervision, Writing – original draft, Writing – review & editing. KE: Conceptualization, Resources, Writing – original draft, Writing – review & editing.
